# Metabolite-mediated crosstalk: unraveling the interactions between gut microbiota and host in fatty liver hemorrhagic syndrome of laying hens

**DOI:** 10.1186/s40104-025-01319-1

**Published:** 2026-01-07

**Authors:** Shaobo Zhang, Xinghua Zhao, Xin He, Wanyu Shi, Ning Ma

**Affiliations:** 1 College of Veterinary Medicine, Veterinary Biological Technology Innovation Center of Hebei Province, Hebei Agricultural University, No.2596, Lekai South Street, Baoding, Hebei Province 071001 China; 2 Present Address: Engineering & Technology Research Center of Traditional Chinese Veterinary Medicine of Gansu Province, Lanzhou Institute of Husbandry and Pharmaceutical Sciences, Chinese Academy of Agricultural Sciences, No. 335, Jiangouyan Street, Lanzhou, Gansu Province 730050 China

**Keywords:** Fatty liver hemorrhagic syndrome, Gut-liver axis, Gut microbiota, Metabolites, Non-alcoholic fatty liver disease

## Abstract

Fatty liver hemorrhagic syndrome (FLHS) in laying hens is a metabolic disorder characterized by excessive hepatic lipid accumulation, inflammation, and hemorrhage, bearing pathological similarities to human non-alcoholic fatty liver disease. With the rise of intensive poultry farming, the incidence of FLHS has markedly increased, resulting in significant economic losses in the poultry industry. The gut microbiota plays a crucial role in host digestion, metabolism, and immune regulation, particularly in liver diseases. Gut microbiota and its metabolites influence liver health via the gut-liver axis. This review aims to explore metabolite-mediated interactions between the laying hens and the gut microbiota, elucidating their role in the pathogenesis of FLHS. Host-derived metabolites, such as lipids, bile acids, amino acids, and carbohydrates, regulate the structure and function of the gut microbiota through the gut-liver axis, playing a role in FLHS progression. Concurrently, microbial metabolites, including short-chain fatty acids, bile acids, and amino acid derivatives, influence hepatic lipid metabolism, inflammation, and oxidative stress, driving the development of FLHS. Key microbes, such as *Bacteroides*, *Lactobacillus*, and *Akkermansia muciniphila*, are considered potential therapeutic targets due to their involvement in metabolite production. By integrating multi-omics data and mechanistic studies, this review highlights the central role of host–gut microbiota communication in FLHS and provides a theoretical basis and research direction for the development of microbiota-based intervention strategies.

## Introduction

Fatty liver hemorrhagic syndrome (FLHS) is a prevalent metabolic disorder in laying hens, marked by hepatic lipid dysregulation, excessive fat deposition, and hemorrhage, resembling human non-alcoholic fatty liver disease (NAFLD) [1]. As intensive poultry farming expands globally, FLHS incidence has surged, posing significant economic challenges to the industry. It is the leading non-infectious disease affecting laying hens, with incidence rates ranging from 5% to 30%, reducing egg production and quality while elevating mortality [2–4]. Studies report mortality rates as high as 74% in some flocks [5], underscoring its severe impact on production efficiency.

**Fig. 1:**
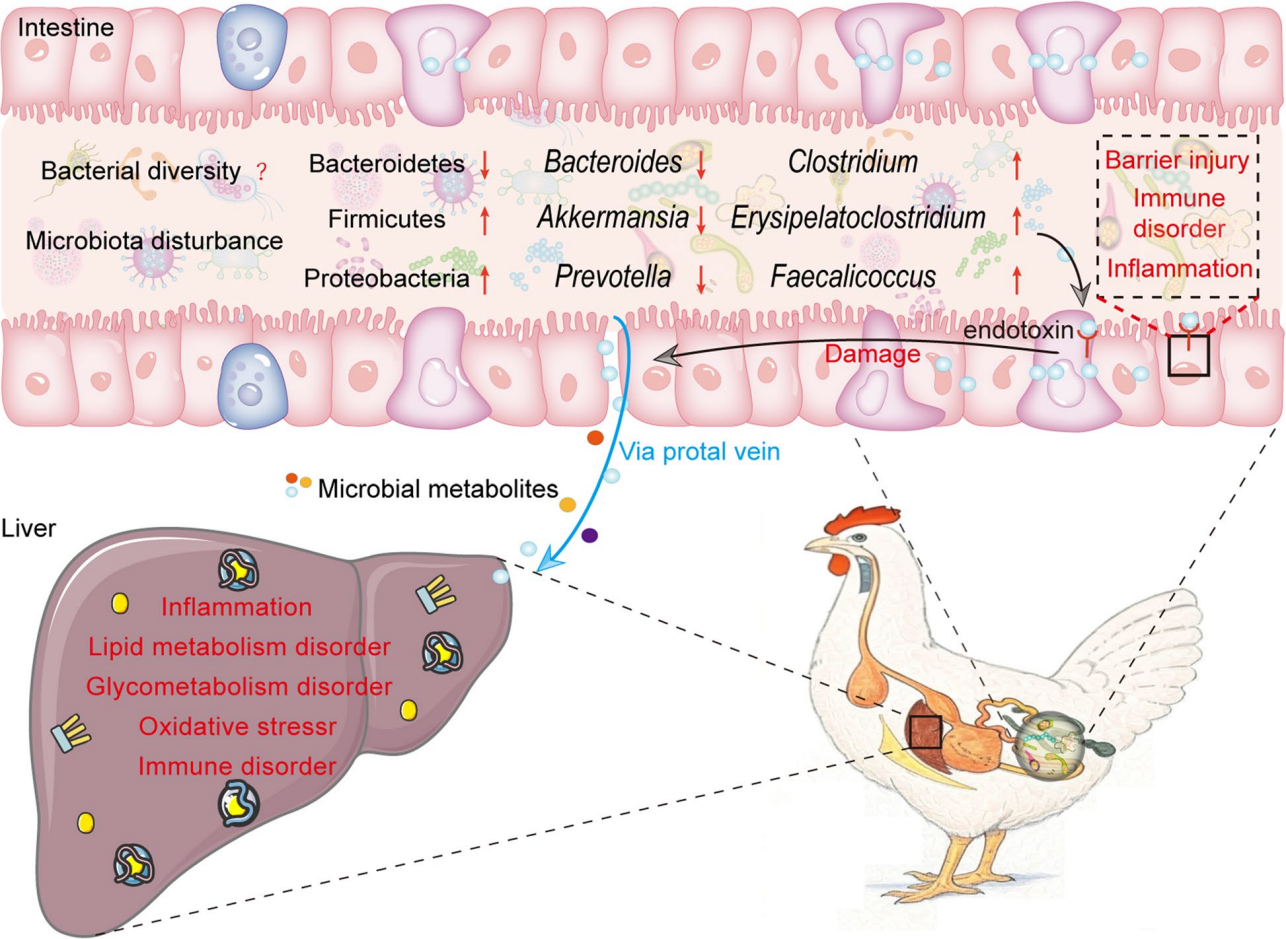
Changes in the structure and function of the gut microbiota in FLHS laying hens and its impact on the liver. The gut microbiota of laying hens affected by FLHS exhibits dysbiosis, characterized by alterations in bacterial abundance manifested as a reduction in beneficial bacteria and an increase in harmful bacterial populations. This microbial imbalance leads to changes in metabolic byproducts, particularly increased production of endotoxins such as LPS, which primarily trigger intestinal inflammation, immune dysregulation, and impairment of the intestinal barrier. The compromised intestinal barrier permits the translocation of harmful substances through the portal vein to the liver, subsequently inducing hepatic lipid metabolism disorders, oxidative stress, and inflammatory responses. FLHS, fatty liver hemorrhagic syndrome; LPS, lipopolysaccharides

The gut microbiota, a complex microbial community in the gastrointestinal tract, is essential for host digestion, metabolism, and immune regulation, with a pronounced role in liver diseases via the gut-liver axis [6, 7]. In FLHS, research consistently shows disrupted gut microbiota (Table 1), leading to intestinal barrier damage [8–17]. This allows endotoxins and metabolites to translocate to the liver, impairing hepatic metabolism, inducing oxidative stress, and triggering inflammation, thus worsening FLHS (Fig. 1) [17–19]. Conversely, a balanced microbiota supports metabolic equilibrium by regulating lipids, amino acids, and bile acids (BAs), mitigating FLHS pathology. Although the link between microbial dysbiosis and FLHS is well-documented, the precise mechanisms involving microbial metabolites remain underexplored. Most studies focus on microbial composition shifts, yet the role of metabolites as mediators between the microbiota and host is critical for understanding FLHS pathogenesis and identifying therapeutic targets.

**Table 1:** Changes in the abundance of gut microbiota in FLHS laying hens

Phylum	Family	Genus	Species
Firmicutes (Cecum) ↑	Lachnospiraceae	*Blautia* (Cecum) *↑*	
		*Flavonifractor* (Cecum) *↓*	
	Erysipelotrichaceae	*Erysipelatoclostridium* (Cecum) ↑	
		*Faecalicoccus* (Cecum) ↑	
	Clostridiaceae	*Clostridium* (Cecum) ↑	
		*Clostridiates *DTU089 (Cecum) *↓*	
	Oscillospiraceae ↑	*Faecalibacterium* (Cecum) ↑	
		*Angelakisella* (Cecum) *↓*	
	Ruminococcaceae	*Ruminococcus* (Cecum) ↑	
	Acidaminococcaceae	*Phascolarctobacterium* (Cecum) *↓*	
	Enterococcaceae	*Enterococcus* (Cecum) ↑	
	Lactobacillaceae	*Lactobacillus* (Cecum) *↓*	*L. aviaries* (Cecum) *↓*
	Selenomonadaceae	*Megamonas* (Cecum) *↓*	
	Veillonellaceae	*Megasphaera* (Cecum) *↓*	
	Turicibacteraceae	*Turicibacter* (Cecum)* ↓*	
	Butyricicoccaceae	*Butyricicoccus* (Cecum) *↓*	
	Monoglobaceae	*Monoglobus* (Cecum) *↓*	
Bacteroidota (Cecum) *↓*	Bacteroidaceae	*Bacteroides* (Cecum) *↓*	*B. salanitronis* (Cecal) *↓*
			*B. caecicola* (Cecum) *↓*
			*B. ndongoniae* (Cecum) *↓*
			*B. togonis* (Cecum)*↓*
	Prevotellaceae	*Prevotella* (Cecum) *↓*	
	Barnesiellaceae (Cecum) ↓	*Barnesiella* (Cecum) *↓*	
	Tannerellaceae	*Parabacteroides* (Cecum) *↓*	
	Odoribacteraceae	*Butyricimonas* (Cecum) *↓*	
Proteobacteria (Cecum) ↑	Sphingomonadaceae	*Sphingomonas* (Ileum) *↓*	
		*Novosphingobium* (Cecum) ↑	
	Comamonadaceae	*Aquabacterium* (Ileum) *↓*	
		*Curvibacter* (Ileum) *↓*	
	Campylobacteraceae	*Campylobacter* (Cecum) ↑	
	Succinivibrionaceae	*Succinatimonas* (Cecum) ↑	
	Pseudomonadaceae	*Pseudomonas* (Ileum) ↑	
	Phyllobacteriaceae	*Phyllobacterium* (Ileum) *↓*	
	Sphaerotilaceae	*Pelomonas* (Ileum) ↑	
	Butyricicoccaceae	*Intestinimonas* (Cecum)↑	
Actinobacteria (Ileum) ↑	Coriobacteriaceae	*Collinsella* (Cecum) *↓*	
	Nocardiaceae	*Rhodococcus* (Ileum) *↑*	
Deferribacterota	Deferribacteraceae	*Mucispirillum* (Cecum) *↑*	*M. schaedleri* (Cecum) *↓*
Thermodesulfobacteriota	Desulfovibrionaceae	*Desulfovibrio* (Cecum) *↑*	*D. piger* (Cecum) *↑*
Verrucomicrobiota	Akkermansiaceae	*Akkermansia* (Cecum)* ↓*	
Methanobacteriota	Methanocorpusculaceae	*Methanocorpusculum* (Cecum) *↓*	
Fusobacteriota	Fusobacteriaceae	*Fusobacterium* (Cecum) *↓*	
Euryarchaeota (Cecum) ↓			
Synergistota (Cecum) ↓			

The upward arrow indicates that the bacteria are upregulated by FLHS (comparing to healthy laying hens), while the downward arrow indicates that the bacteria are downregulated by FLHS [9–16]

Metabolites facilitate a bidirectional interaction between the gut microbiota and host through the gut-liver axis. Host-derived metabolites, such as fatty acids and BAs, modulate microbial structure and function [20]. In FLHS, altered hepatic BA biosynthesis influences intestinal microbiota composition, exacerbating disease progression. Meanwhile, microbial metabolites (short-chain fatty acids (SCFAs), BAs, and amino acids) enter the liver via enterohepatic circulation, regulating lipid metabolism, inflammation, and oxidative stress [9]. For instance, lactate bacteria produce organic acids and amino acids that stabilize the intestinal microenvironment and support hepatic balance [16, 21]. Key microbes, including *Bacteroides*, *Parabacteroides*, and *Akkermansia muciniphila*, are noted for their metabolite-mediated effects on hepatic lipid and energy metabolism [22–24]. Thus, both host-derived and bacteria-derived metabolites participate in the bidirectional interaction between the gut microbiota and FLHS via the gut-liver axis. While most current research has focused on the influence of the gut microbiota on the host, the reciprocal regulation by host metabolites on the composition and function of the gut microbiota, and how this bidirectional interaction drives the progression of FLHS, remains insufficiently explored.

This review aims to elucidate the interaction mechanisms between the gut microbiota and host in FLHS, focusing on metabolites as central communicators. Host metabolites can affect FLHS by regulating the composition of the gut microbiota. Alternatively, microbiota-derived metabolites may regulate hepatic metabolism to participate in the pathogenesis of FLHS. By integrating multi-omics data and mechanistic insights, the review highlights how metabolites bridge microbial ecology and laying hens' metabolic health. This work provides novel insights into FLHS pathogenesis. Furthermore, it establishes a theoretical basis for microbiota-based interventions to prevent or treat FLHS. Such interventions could include targeted modulation of microbial communities or metabolites, ultimately enhancing laying hen health and productivity.

## Lipid-mediated interactions between the gut microbiota and the host in FLHS

Fatty liver hemorrhagic syndrome in laying hens is a condition fundamentally characterized by severe lipid metabolism disorders. Lipids and their metabolic pathways play a central role in the pathogenesis of FLHS, leading to abnormal lipid accumulation, liver dysfunction, and hemorrhage [25–27]. In FLHS, dysregulated lipid metabolism alters the composition of lipids excreted into the intestine via bile, thereby shaping the structure and function of the gut microbial community through the gut-liver axis [25, 26, 28]. In turn, the gut microbiota produces a range of lipid metabolites that can either mitigate or exacerbate FLHS progression. This intricate bidirectional relationship underscores the complexity of FLHS and highlights the significance of lipids, as understanding their role may pave the way for novel lipid-based therapeutic strategies to manage or prevent this syndrome in laying hens. This section explores how host-derived lipids influence the role of gut microbiota in FLHS regulation, as well as how microbial lipid metabolites modulate FLHS through the gut-liver axis.

### Effects of host-derived lipids on gut microbiota composition in FLHS

Only a small portion of the lipids produced by liver metabolism is excreted into the intestine via bile, thereby affecting the gut microbiota. Most lipids do not directly enter the gut. Among them, phosphatidylcholine (PC) is the main bile component secreted by the liver and is the only lipid affected by FLHS that can reach the gut [29]. PC plays a key role in fat digestion and absorption and significantly influences the composition and metabolic activity of the gut microbiota.

After entering the intestine, PC helps emulsify and digest fats as a major component of bile [30]. In addition, PC serves as a nutrient for certain beneficial bacteria, which helps maintain gut microbiota diversity and stability [31, 32]. Studies show that PC intake is closely related to changes in the abundance of specific bacterial species. For example, *Lactobacillus* and *Alistipes* can use PC as a nutrient, and their abundance increases when PC intake is high [33]. These bacteria are often associated with gut health, immune regulation, and metabolic balance [33, 34]. Conversely, PC intake can suppress the abundance of potentially harmful bacteria such as *Faecalibaculum*, *Dubosiella*, *Turicibacter*, and *Parasutterella*, whose overgrowth is linked to gut inflammation and FLHS [31–33]. Dietary PC supplementation has been shown to reverse high-fat diet-induced obesity and insulin resistance (IR), an effect that is closely related to improvements in gut microbiota structure [34]. For example, PC rich in eicosapentaenoic acid and docosahexaenoic acid can maintain the abundance of beneficial bacteria such as Bacteroidetes and *Akkermansia* while suppressing potentially harmful Firmicutes, thereby improving gut ecological balance [34]. By regulating gut microbiota diversity and the abundance of specific species, PC further influences host lipid metabolism, intestinal barrier function, and immune responses, highlighting its potential therapeutic value in metabolic diseases such as obesity, IR, and NAFLD.

A substantial proportion of host-derived lipids do not directly reach the intestinal lumen. However, they can still indirectly modulate the gut microbial community. This indirect regulation may occur through the secretion of lipid components in bile or via dietary supplementation of specific lipids that are altered in FLHS. Interestingly, supplementing the lipids that are reduced in FLHS can improve the condition by modulating the gut microbiota. For example, as mentioned earlier, supplementing with PC improves hepatic steatosis by altering the gut microbiota. In FLHS laying hens, the levels of arachidonic acid are decreased in both the liver and the gut [16, 24, 35]. Research has shown that adding arachidonic acid can modulate the gut microbiota in a sex-dependent manner to improve fatty liver disease [36]. In males, supplementation with arachidonic acid triggers gut microbiota-driven inflammation that accelerates the progression of fatty liver, while in females it adjusts the inflammation associated with the microbiota, thereby ameliorating fatty liver. In FLHS laying hens, supplementing arachidonic acid has also been shown to reduce hepatic inflammation and improve FLHS [24]. Additionally, dietary docosahexaenoic acid-phosphatidylserines can lower the ratio of Firmicutes to Bacteroidetes, reduce the abundance of *Lachnoclostridium*, Coriobacteriaceae, Desulfovibrionaceae, and *Helicobacter*, and increase the levels of *Lachnospiraceae_NK4A136_group*, *Alistipes*, *norank_f_Muribaculaceae*, and *Bacteroides*, thereby improving jejunal injury and NAFLD induced by a high-fat diet [37, 38]. Lipid homeostasis is a crucial indicator of liver health, so supplementing the lipids that are compromised under pathological conditions may represent a promising therapeutic strategy for FLHS.

### Regulatory roles of gut microbiota-mediated lipid metabolites in FLHS

#### Lipid metabolites produced by the gut microbiota

The gut microbiota ferments dietary fiber, degrades host metabolites, and synthesizes its own membrane lipids, producing a variety of lipid metabolites. These include: (1) SCFAs, produced by different bacteria via specific pathways. Acetate is mainly generated by *Bacteroides* (via glycolysis), *Bifidobacterium* (via the pentose phosphate pathway), and *A. muciniphila* (via mucin degradation) [39–41]; propionate is produced by *Prevotella* (via the succinate-propionate pathway) and *Propionibacterium* (via the Wood-Werkman cycle) [42]; butyrate is synthesized by *Roseburia* (via the butyrate kinase pathway) and *Faecalibacterium prausnitzii* (via the acetyl-CoA pathway) [40]. (2) Polyunsaturated fatty acids, such as arachidonic acid produced by *Bacteroides fragilis*, and conjugated linoleic acid (CLA) produced by *Bifidobacterium* and *Lactobacillus* through CLA-hydratase enzyme activity. Both arachidonic acid and CLA show potential in reducing inflammation and improving lipid metabolism [24, 43]. (3) Membrane lipids, such as phosphatidylethanolamine and sphingolipids, which may participate in metabolic regulation through colocalization with host tissues [44]. (4) Glycolipids, such as LPS produced by Gram-negative bacteria (e.g., *E. coli*), which are closely related to inflammatory responses [45]. The abundance and types of these metabolites directly shape the gut ecosystem and significantly affect liver health through the gut-liver axis, especially in FLHS (Fig. 3A).

#### Effects of SCFAs on FLHS

Short-chain fatty acids, such as acetate, propionate, and butyrate, are key metabolites produced from dietary fiber fermentation by the gut microbiota and exert a multi-faceted protective role in FLHS through the gut-liver axis.

Short-chain fatty acids directly modulate lipid homeostasis in the liver. They activate the adenosine 5'-monophosphate-activated protein kinase (AMPK) signaling pathway, which in turn upregulates genes essential for fatty acid β-oxidation (e.g., carnitine palmitoyltransferase 1 A and acyl-CoA oxidase 1) and concurrently inhibits key lipogenic enzymes (e.g., acetyl CoA carboxylase and fatty acid synthase) [46, 47]. Furthermore, SCFAs enhance the expression of peroxisome proliferator-activated receptor alpha (PPARα) while suppressing sterol regulatory element-binding protein-1c (SREBP-1c), a master regulator of de novo lipogenesis [48]. Collectively, these actions reduce hepatic triglyceride (TG) accumulation by promoting fat oxidation and curbing fat synthesis. For instance, acetate is known to lower liver TG levels by activating AMPK [49] while propionate can activate the adiponectin-AMPK-PPARα pathway to directly improve fatty liver [12]. Propionate also improves insulin sensitivity and contributes to gluconeogenesis and cholesterol metabolism, indirectly alleviating the liver's lipid burden [50].

Short-chain fatty acids are potent immunomodulators. By binding to G-protein-coupled receptors 41 and 43, they can suppress the nuclear factor kappa-B (NF-κB) pathway, leading to a reduced release of proinflammatory cytokines like TNF-α and IL-6, thereby alleviating hepatic inflammation [51]. Concurrently, SCFAs activate the nuclear factor erythroid 2-related factor 2 pathway, which upregulates a suite of antioxidant enzymes. This dual action mitigates oxidative stress and lessens the overall inflammatory burden on the liver [52, 53].

Among SCFAs, butyrate is particularly crucial for gut health. It serves as the primary energy source for enterocytes, thereby strengthening the intestinal barrier function. A robust barrier limits the translocation of endotoxins (like LPS) from the gut to the liver, further protecting the liver from inflammatory triggers [54, 55]. Experimental studies in FLHS models confirm that butyrate supplementation significantly reduces liver TG levels, steatosis, and inflammatory infiltration [54]. Beyond its barrier-protective and anti-inflammatory roles, sodium butyrate has also been shown to modulate crucial cellular processes in hepatocytes of FLHS hens, including improving autophagy and mitigating ferroptosis and apoptosis [56, 57].

These findings underscore the significant therapeutic potential of SCFAs in FLHS intervention. To translate these insights into practical applications, future strategies should focus on optimizing SCFA production and delivery. Approaches such as leveraging prebiotics or probiotics to enhance endogenous SCFA generation, or developing targeted supplementation methods (e.g., coated butyrate derivatives), could amplify their protective effects. Such methods hold promise for boosting SCFA bioavailability and could be developed into effective, targeted flock-level health strategies.

#### Beneficial and detrimental lipid metabolites from the microbiota

Besides SCFAs, other complex lipid metabolites produced by the gut microbiota have dual effects in FLHS. CLA is produced from linoleic acid by *Bifidobacterium* and *Lactobacillus* through CLA-hydratase enzyme activity. CLA binds to G protein-coupled receptor 40 and G protein-coupled receptor 120, thereby trigging anti-inflammatory signals and limiting proinflammatory lipid production. This process significantly reduces liver TG levels and improves lipid metabolic disorders in FLHS hens [58]. Polyunsaturated fatty acids such as arachidonic acid, produced by *B. fragilis*, activate the PPARγ pathway to enhance fatty acid oxidation and inhibit lipid synthesis [24]. This reduces liver fat load and alleviates oxidative stress through its anti-inflammatory properties. Moreover, membrane lipids synthesized by Bacteroidetes, such as phosphatidylethanolamine and sphingolipids, can be transferred to host tissues via outer membrane vesicles [59]. This process helps regulate intestinal barrier function and immune responses, indirectly reducing harmful metabolite translocation to the liver and alleviating FLHS pathology. Lipids produced by the gut microbiota can not only directly improve fatty liver but also indirectly enhance liver health through bacterial cross-feeding. *Bacteroides*, which possess a robust polysaccharide degradation system, are considered key species within the microbial community. For example, *Bacteroides uniformis* utilizes its polysaccharide utilization loci to cross-feed butyrate-producing Firmicutes, which leads to elevated butyrate levels and an improvement in hepatic steatosis [60]. These protective lipid metabolites offer potential targets for FLHS intervention.

However, some bacterial lipid metabolites may worsen FLHS. Glycolipids like LPS, produced by Gram-negative bacteria (like *E. coli*), can enter the liver through the gut-liver axis when the intestinal barrier is impaired in FLHS. LPS activates the Toll like receptor 4 pathway, induces the release of proinflammatory cytokines (such as TNF-α and IL-1β), and aggravates liver inflammation and lipid deposition, contrasting with the protective effects of SCFAs [61]. In addition, bacterial glycolipids may be presented by CD1d cells and recognized by natural killer T cells, triggering inflammatory damage in the liver [62]. This dual effect highlights the complexity of FLHS pathology. While current studies have elucidated some mechanisms underlying lipid metabolic alterations, there are still significant gaps. First, comprehensive lipidomics analyses are lacking. Existing research does not cover the full spectrum of lipid metabolites related to the gut microbiota in FLHS, especially for key bacteria such as *Bacteroides*, *Akkermansia*, *Bifidobacterium*, and *Lachnospira*, whose metabolites significantly affect laying hen health. Second, there is insufficient research on the microbial enzymes that mediate lipid metabolism. Limited progress in genetic manipulation and regulatory approaches restricts precise intervention in microbial lipid pathways.

## Bile acids-mediated communication between the gut microbiota and the host in FLHS

Bile acids, the end products of hepatic cholesterol metabolism, are chemically defined as steroid lipids. Beyond their classical role in fat digestion, they also act as critical signaling molecules that regulate host metabolism, inflammation, and immune responses, as well as shape the gut microbial community. Due to this distinct and pivotal role in the gut-liver axis, particularly in FLHS, this section discusses their mediated interactions separately from other lipid classes.

**Fig. 2:**
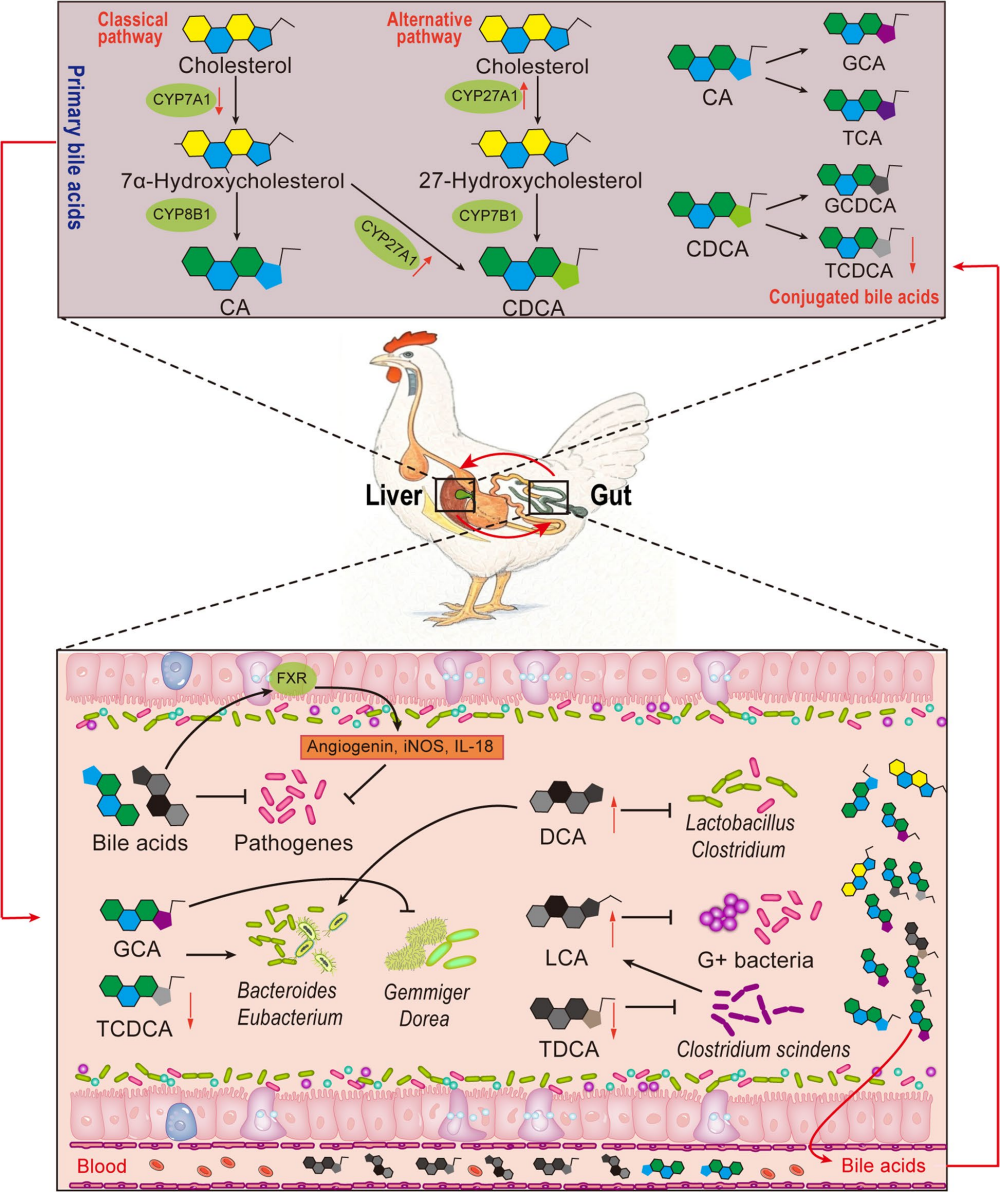
Changes in bile acid metabolism in FLHS laying hens and its impact on the gut microbiota. In FLHS, the classic pathway for bile acid synthesis in the liver is impeded, while the alternative pathway is enhanced. Bile acids synthesized in the liver are excreted into the intestine via the bile duct. They can activate the FXR, which in turn increases the secretion of angiogenin, iNOS, and IL-18, thereby exerting antibacterial effects. Bile acids themselves possess antibacterial properties, with different types exhibiting specific antibacterial spectra. Furthermore, in bacteria that are resistant to bile acids or utilize bile acids as nutrients, bile acids selectively promote their growth. Ultimately, bile acids produced in the intestine are reabsorbed by the intestinal epithelium, enter the bloodstream, and then return to the liver, thereby completing the enterohepatic circulation of bile acids. The black arrow in the figure indicates promoting effect, and "⊣" indicates inhibitory effect; An upward red arrow indicates an increase in content, while a downward red arrow indicates a decrease in content. FLHS, fatty liver hemorrhagic syndrome; FXR, farnesoid X receptor; IL-18, interleukin 18; CYP7A1, cholesterol 7α-hydroxylase; CYP8B1, sterol 12α-hydroxylase; CYP27A1, sterol 27-hydroxylase; CYP7B1, cholesterol 7α-hydroxylase; CA, cholic acid; CDCA, chenodeoxycholic acid; GCA, glycocholic acid; TCA, taurocholic acid; GCDCA, glycochenodeoxycholic acid; TCDCA, taurochenodeoxycholic acid; DCA, deoxycholic acid; LCA, lithocholic acid; TDCA, taurodeoxycholic acid

Bile acids are the end products of hepatic cholesterol metabolism, playing a fundamental role in fat digestion and absorption while also acting as critical signaling molecules. By activating nuclear and membrane receptors, BAs regulate host lipid metabolism, energy homeostasis, and immune responses [63]. In FLHS, liver dysfunction and intestinal dysbiosis lead to disruption of BA metabolism. Consequently, BA-mediated interactions between the gut microbiota and the host become particularly prominent [64]. Generally, the host can directly synthesize primary BAs, so they are primarily considered to be host-derived. Secondary BAs are microbiota-modified molecules derived from host-synthesized primary BAs. However, given the critical role of BAs in enterohepatic circulation, secondary BAs are also recognized as having a host-derived component. This section reviews how host-derived BAs shape the gut microbial community and how microbial modifications of BAs influence FLHS pathology through the gut-liver axis (Fig. 2).

### Host-derived BAs regulate gut microbiota in FLHS

Alterations in the concentrations and composition of host-secreted primary BAs, along with their impacts on signaling pathways, can reshape the gut microbiota. This creates a bidirectional network between the microbiota and the host that significantly affects FLHS pathology [65].

Firstly, BAs have antimicrobial properties and can selectively inhibit the growth of certain bacteria [66]. High concentrations of BAs inhibit the proliferation of Gram-negative bacteria (such as Proteobacteria) and reduce endotoxin (e.g., LPS) release, thereby lessening liver inflammation. However, excessive BAs may also reduce microbial diversity [67]. Low BA concentrations facilitate the proliferation of commensal microbiota (particularly *Bacteroides* and *A. muciniphila*), while concurrently exerting dual regulatory effects via farnesoid X receptor (FXR)-mediated transcriptional regulation of antimicrobial peptides to concomitantly inhibit pathogenic overgrowth, thereby preserving intestinal homeostasis [65, 68]. Additionally, primary BAs have inhibitory effects on sensitive species in Bacteroidetes and Firmicutes, while secondary BAs like deoxycholic acid (DCA) and lithocholic acid (LCA) more strongly inhibit Gram-positive bacteria [69, 70]. After feeding DCA, the abundance of *Bacteroides* and *Parabacteroides* in the intestine increases, while the levels of bacteria such as *Lactobacillus*, *Clostridium XI*, and *Clostridium XIV* decrease significantly [71]. In contrast, LCA exhibits strong inhibitory effects on bacteria including *Staphylococcus aureus*, *E. coli*, and *Bacillus cereus* [72]. In FLHS, abnormal BA profiles (reduced primary BAs and increased secondary BAs) disturb gut microbiota balance. This may lead to a decline in beneficial bacteria (such as *Lactobacillus* and *Bifidobacterium*) and an increase in opportunistic pathogens (such as *E. coli* and *Enterococcus*), worsening intestinal barrier damage and endotoxin translocation, and further aggravating liver inflammation [65].

Secondly, conjugated BAs exerts microbiome-shaping effects through selective pressure. Specifically, taurine-conjugated BAs (such as taurochenodeoxycholic acid and taurocholate acid) selectively promote the growth of *Bacteroides* whose metabolite succinate can activate intestinal gluconeogenesis and improve IR [73]. Taurodeoxycholic acid reduces the abundance of *Clostridium scindens*, thereby decreasing the production of LCA [74]. Glycine-conjugated BAs (such as glycocholic acid) may promote bacteria such as *Bacteroides* and *Eubacterium* while inhibiting *Gemmiger* and *Dorea*. This change increases SCFA levels, improves mitochondrial function, reduces oxidative stress, and lowers inflammation risk, thereby regulating liver lipid metabolism [75, 76].

Moreover, host BAs indirectly regulate the gut microbiota through FXR-mediated communication. FXR activation enhances the expression of tight junction proteins in intestinal epithelial cells, strengthens barrier function, and reduces LPS leakage. It also upregulates fibroblast growth factor (FGF) 15/19 expression, which feeds back to suppress hepatic cholesterol 7α-hydroxylase expression and reduce BA synthesis [77]. In FLHS, reduced FXR signaling in both the liver and intestine leads to gut microbiota dysbiosis (lower *Ruminococcus* and higher *Desulfovibrio*), intestinal permeability increased, and BA metabolism and gut leakiness worsened [78, 79]. BAs also influence microbial metabolic activity by regulating intestinal pH and BA-binding proteins (such as apical sodium-dependent BA transporter) [80]. The changes in FXR and FGF signaling reshape the gut microbiota. Subsequently, microbial metabolites provide feedback that affects host metabolic homeostasis. This highlights the multidimensional role of BAs in regulating the gut microbiota.

### Role of microbiota-derived BAs on FLHS

**Fig. 3:**
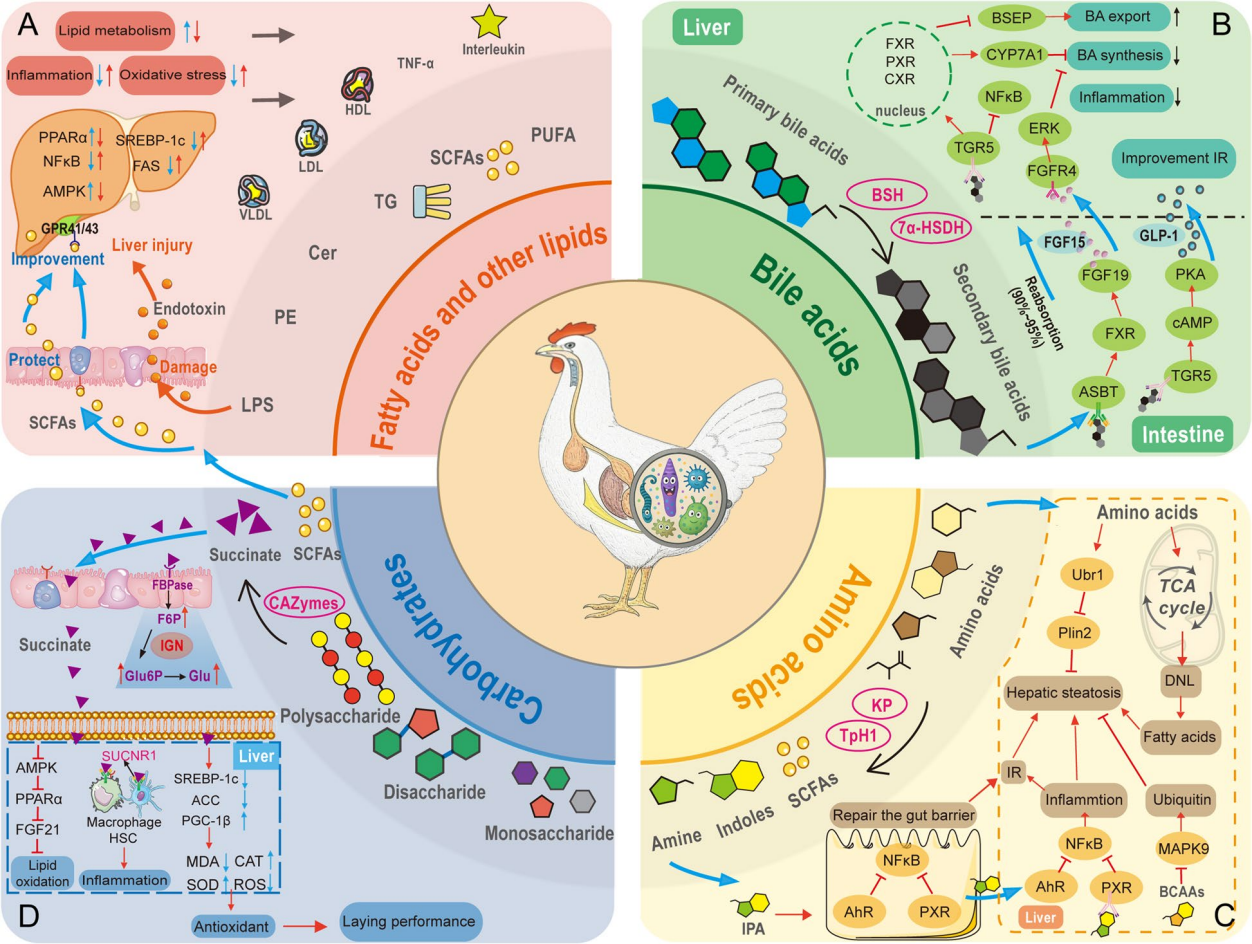
Mechanisms of action of gut microbiota-derived metabolites on FLHS. **A** Mechanisms of lipids and fatty acids on FLHS based on the gut-liver axis. Blue arrows show that gut microbiota-derived SCFAs bind to receptors (e.g., GPR41/43) in the gut and liver, suppressing inflammation, reducing oxidative stress, and enhancing lipid metabolism. This lowers liver and blood lipids and inflammatory factors. Red arrows indicate that endotoxins produced by the gut microbiota initially damage the intestinal epithelial barrier, increasing its permeability. This damage allows a significant amount of endotoxin to transfer from the gut to the liver, causing hepatic injury. **B** Mechanism of BAs in FLHS. Gut microbiota converts primary BAs to secondary BAs using enzymes like BSH and 7α-HSDH. Most of BAs are reabsorbed in the ileum and transported to the liver, binding to TGR5 to reduce inflammation and to FXR and PXR to regulate BA synthesis and export. In the gut, secondary BAs bind ASBT, enter intestinal cells, and activate FXR to increase FGF 15/19 expression. FGF15/19 travels to the liver, binds FGFR4, and inhibits BA synthesis via the ERK pathway. **C** Amino acid metabolism in FLHS. Gut microbiota metabolizes amino acids into derivatives like SCFAs, indoles, and BCAAs via enzymes such as KP and TpH1. These derivatives activate E3 ubiquitin ligase (Ubr1), promoting Plin2 degradation to reduce hepatic lipid degeneration. The tryptophan derivative IPA activates aryl hydrocarbon receptor and PXR, inhibiting NF-κB to reduce gut inflammation and repair the intestinal barrier. BCAAs inhibit MAPK9 and enhance ubiquitination, alleviating steatosis. **D** Carbohydrate metabolism in FLHS. Gut microbiota hydrolyzes carbohydrates via CAZymes into SCFAs and succinate. Succinate binds FBPase, elevating F6P and Glu6P levels, enhancing intestinal gluconeogenesis. In the liver, succinate inhibits lipid oxidation by suppressing the AMPK/PPARα/FGF21 pathway and activates SUCNR1 on macrophages and hepatic stellate cells, promoting inflammation. It enhances lipid synthesis and antioxidant capacity, improving FLHS laying performance. FLHS, fatty liver hemorrhagic syndrome; SCFAs, short-chain fatty acids; BAs, bile acids; BSH, bile salt hydrolase; 7α-HSDH, 7α-hydroxysteroid dehydrogenase; TGR5, G protein-coupled bile acid receptor 1 FXR, farnesoid X receptor; PXR, pregnane X receptor; ASBT, apical sodium-dependent BA transporter; FGF, fibroblast growth factor; ERK, extracellular regulated protein kinases; PKA, protein kinase A; BCAAs, branched-chain amino acids; KP, kynurenine pathway; TpH1, tryptophan hydroxylase 1; AhR, aryl hydrocarbon receptor; IPA, indole-3-propionic acid; MAPK9, mitogen-activated protein kinase 9; FBPase, fructose 1,6-bisphosptase; F6P, fructose 6-phosphate; Glu6P, glucose-6-phosphatase G-6-pase; PPARα, perixisome proliferation-activated receptor alpha; SUCNR1, succinate receptor 1 curated

The gut microbiota converts primary BAs into secondary BAs (such as DCA, LCA, and ursodeoxycholic acid) via dehydroxylation and desulfation. These metabolites return to the liver through the gut-liver axis and influence FLHS pathology (Fig. 3B) [81]. Microbial enzymes play a key role in this process. Bile salt hydrolase (BSH), expressed by bacteria such as *Lactobacillus* and *Bifidobacterium*, hydrolyzes conjugated BAs to release free BAs [82]. This process provides substrates for further microbial metabolism (like dehydroxylation), enhances FXR signaling, and suppresses SREBP-1c, which reduces liver fat deposition [83, 84]. The enzyme 7α-dehydroxylase, expressed by bacteria such as *Clostridium scindens*, converts CA into DCA. A lack of this enzyme is correlated with lower DCA levels in FLHS hens [85]. In FLHS, changes in BSH activity directly affect the efficiency of secondary BA production and the composition of the BA pool [86]. For example, enhanced BSH activity may increase free BAs, which are then converted by the microbiota into DCA and LCA. These metabolites activate FXR and the G protein-coupled bile acid receptor 1 (TGR5) signaling pathways to regulate liver lipid metabolism and inflammation [82]. Conversely, reduced BSH activity may decrease secondary BA production, weakening their regulatory effect on liver metabolism. Studies have shown that in fatty liver disease, abnormal BSH activity and subsequent DCA accumulation are associated with increased oxidative stress and liver damage, highlighting the dual effects of BSH in disease progression [87, 88].

The anti-inflammatory and lipolytic effects of secondary BAs differ between healthy and pathological states. In healthy conditions, secondary BAs activate FXR and TGR5 to inhibit lipid accumulation and inflammation. For example, hyodeoxycholic acid has anti-inflammatory and hepatoprotective effects by upregulating PPARα and carnitine palmitoyltransferase 1 A, promoting fatty acid β-oxidation, and reducing hepatocyte steatosis and oxidative stress [89, 90]. DCA stimulates intestinal endocrine cells to secrete glucagon-like peptide-1 via the TGR5-cyclic adenosine monophosphate-protein kinase A pathway, thereby improving insulin sensitivity [91, 92]. However, in FLHS, gut dysbiosis leads to reduced production of protective BAs such as hyodeoxycholic acid, which weakens liver protection [93]. In contrast, high concentrations of DCA and LCA may induce oxidative stress and inflammation, worsening liver damage [94, 95]. For example, studies show that DCA accumulation in fatty liver patients is correlated with increased liver inflammatory cytokines (such as TNF-α), suggesting that the effects of secondary BAs depend on their type and concentration [96].

Intervention studies further reveal the therapeutic potential of microbiota-derived BAs. Supplementing specific BAs (such as taurine deoxycholic acid) can improve liver lipid metabolism in FLHS hens and reduce steatosis and inflammatory infiltration [97, 98]. This may be achieved by enhancing FXR signaling, inhibiting cholesterol 7α-hydroxylase expression, and maintaining cholesterol homeostasis. In older laying hens, supplementing with porcine BAs can enrich beneficial bacteria in the cecum, thereby regulating blood lipid metabolism, improving liver steatosis, and enhancing production performance [88]. Moreover, probiotics (such as *Clostridium butyricum*) can reshape the gut microbiota and BA profile, increasing levels of tauroursodeoxycholic acid and LCA, which significantly lower liver fat deposition [99]. Mechanistically, secondary BAs regulate liver gene expression via the gut-liver axis. In addition, BAs such as 3-succinylated BA produced by *B. uniformis* promote the growth of *A. muciniphila*, which in turn inhibits liver inflammation and steatosis (with 3-succinylated BA levels in metabolic-dysfunction-associated fatty liver disease patients negatively correlated with liver damage) [87]. *Christensenella minuta* produces 3-O-acyl BAs that improve IR and liver lipid metabolism by inhibiting intestinal FXR [100]. These studies revealed the beneficial effects of microbiota-derived BAs on FLHS. However, as previously mentioned, high concentrations of DCA and LCA can cause liver damage through pathways including mitochondrial dysfunction, inflammation, cell death, and DNA damage [94, 95]. These findings indicate that bacterial BA metabolites may act as either protective or harmful factors in FLHS, depending on microbial activity and the dynamic balance of the BA pool.

Despite advances in understanding BA-mediated interactions in FLHS, several research gaps remain. The precise microbial species and enzymes responsible for BA modifications in FLHS are underexplored. Future research should focus on mapping the detailed BA metabolic pathways in FLHS and identifying key microbial contributors. Exploring targeted interventions, such as probiotics or BA supplements, could provide strategies to restore BA balance and alleviate FLHS symptoms. Further investigation into the molecular mechanisms by which microbial BA metabolites affect liver function and inflammation is also essential for developing effective therapeutic approaches.

## Amino acid-mediated interactions between the gut microbiota and the host in FLHS

Beyond the intricate signaling networks of lipids and BAs, disturbances in amino acid metabolism represent another critical layer of host-microbiota crosstalk that profoundly influences the pathogenesis of FLHS. Amino acids serve not only as the basic building blocks for protein synthesis but also play crucial roles in metabolic regulation, immune responses, and maintaining microbial ecological balance. Recent studies have shown that disruptions in amino acid metabolism are closely linked to metabolic liver diseases such as FLHS and NAFLD [101]. In FLHS, the amino acid metabolic profile is markedly abnormal, with decreased levels of branched-chain amino acids (BCAAs) such as valine, leucine, and isoleucine, and elevated concentrations of aromatic amino acids like phenylalanine and tyrosine, as well as sulfur-containing amino acids such as methionine and cysteine [101–103]. These imbalances exacerbate hepatic fat deposition, inflammation, and oxidative stress, contributing to the progression of FLHS. Amino acids orchestrate hepatic metabolic reprogramming through microbiota-dependent modulation of the gut-liver axis, thereby regulating FLHS progression. This section explores how host amino acids influence the gut microbiota and how microbial amino acid metabolites impact FLHS, highlighting the intricate interplay between amino acid metabolism and microbial activity in the context of liver health.

### Amino acids modulate gut microbiota in FLHS

Amino acids act as key nutrients and signaling molecules for the gut microbiota, significantly regulating its composition and metabolic activity. Host-produced amino acids cannot exert a direct effect on the gut microbiota. Nevertheless, deficiency or excessive intake of certain amino acids in the diet can still impact the gut microbiota and contribute to the progression of FLHS. A deficiency in BCAAs can inhibit the growth of beneficial bacteria such as *Akkermansia* and *Bifidobacterium*, leading to a reduction in SCFA production and weakened intestinal barrier function [104]. Conversely, an excess of aromatic amino acids provides substrates for pathogenic bacteria like *E. coli*, promoting the production of pro-inflammatory metabolites such as phenylacetic acid and p-cresol sulfate, which activate the NF-κB pathway and disrupt tight junction proteins [105]. Methionine deficiency reduces glutathione levels, thereby exacerbating intestinal oxidative stress and hindering the colonization of beneficial species such as *F. prausnitzii* [106, 107]. Supplementation with lysine can upregulate the abundance of *Lactobacillus* and *Dorea* and increase the production of anti-inflammatory metabolites, indirectly improving hepatic lipid metabolism [108]. Long-term high-valine diets have been associated with an increased abundance of Fusobacteriota and Deferribacterota, triggering inflammation through the general control nonderepressible 2 pathway and suppressing fatty acid oxidation [103].

Dietary amino acid supplementation has the potential to improve metabolic diseases by modulating the gut microbiota. Oral administration of BCAAs can enhance microbial diversity, increase the abundance of *Bacteroides*, and suppress pathogens such as *Campylobacter*, *Mitsuokella*, and *Pascolarctobacterium*, thereby ameliorating hepatic steatosis [109]. Taurine supplementation has been found to increase the levels of *A. muciniphila*, strengthen the intestinal barrier, and reduce FLHS-related liver damage [26, 110]. Additionally, serine and glycine promote the growth of *Lactobacillus* and inhibit pathogens, thereby optimizing the intestinal microecology [111]. Dimethylglycine can increase the abundance of *Staphylococcus* and boost β-glucuronidase activity, leading to higher circulating estrogen levels, improved intestinal barrier function, and alleviation of FLHS [112]. Glycine supplementation also reduces stress hormone levels and inhibits the proliferation of Proteobacteria, further improving intestinal barrier integrity [113].

Although a link between amino acid metabolism and FLHS is confirmed, many questions remain. The dominant metabolites driving the disease and their precise molecular mechanisms are still unclear. Furthermore, while interventions like amino acid supplementation show promise, their long-term safety, optimal dosage, and timing require rigorous validation through large-scale experiments to avoid potential side effects. Future research integrating multi-omics approaches is essential to systematically uncover these regulatory networks and develop effective, targeted intervention strategies.

### Regulation of microbial amino acid metabolites on FLHS

The gut microbiota metabolizes amino acids through decarboxylation, deamination, and fermentation to produce various bioactive compounds, including indole derivatives, amines, BCAAs, SCFAs, and hydrogen sulfide [114]. These amino acids and metabolites enter the liver via the gut-liver axis, where they regulate the expression of key genes and metabolic pathways, significantly influencing FLHS progression (Fig. 3C).

#### Indole derivatives

Gut bacteria convert tryptophan into indole derivatives such as indole-3-carboxylic acid, kynurenine, and other tryptamine compounds, which possess anti-inflammatory and antioxidant properties [115]. In FLHS laying hens, reduced levels of these indole derivatives are associated with dysbiosis and increased hepatic oxidative stress. Indole-3-propionic acid, produced by *Clostridium sporogenes* from tryptophan, activates the pregnane X receptor to inhibit the NF-κB pathway and downregulate lipogenic genes, thereby reducing liver inflammation and steatosis [116]. *Bacteroides coccoides* can metabolize tryptophan into indole-3-acetic acid, which activates hepatic aryl hydrocarbon receptor to enhance insulin sensitivity and reduce body fat in animal models [117]. Supplementation with *Hericium erinaceus* or magnolol increases the abundance of *Lactobacillus*, elevates the production of tryptophan metabolites, and activates the aryl hydrocarbon receptor pathway to markedly alleviate hepatic inflammation [118, 119]. Similarly, adding glucomannan boosts *Bacteroides ovatus* levels, and enhances intestinal barrier function by activating the aryl hydrocarbon receptor through its metabolite indoleacetic acid, thereby ameliorating IR [120]. This exerts a positive impact on hepatic glucose and lipid metabolism.

#### Amines

Amines such as histamine, spermidine, and tyramine, produced by microbial metabolism of amino acids, have dual roles in immune regulation and maintenance of the intestinal barrier. In FLHS, the abnormal accumulation of these amines may worsen intestinal inflammation, and their transport via the portal vein can trigger inflammatory cascades and lipid peroxidation in the liver [121]. Excessive production of tyramine from tyrosine metabolism may activate the Toll like receptor 4 pathway, thereby exacerbating liver injury [122].

#### Branched-chain amino acids

The changes in BCAA levels remain controversial in fatty liver disease. High doses of BCAAs can reduce hepatic lipid synthesis and enhance fatty acid β-oxidation, thereby improving FLHS pathology [102]. Additionally, BCAAs and their metabolites may attenuate the phosphatidylinositol 3-kinase/protein kinase B signaling pathway in the liver and other tissues, which is considered a major mechanism for their beneficial effects beyond mere nutrition [123]. On the other hand, elevated circulating BCAA levels are associated with higher cholesterol, increased liver fat, and IR [124]. Resistant starch has been shown to alleviate hepatic steatosis by reducing the abundance of *Bacteroides stercoris* and subsequently lowering BCAA production, particularly valine [125]. The relationship between dietary BCAAs, circulating levels, and FLHS exhibits intricate complexity with apparent dual effects. The biological functions of BCAAs diverge across pathological states, involving multifaceted mechanisms such as gut microbiota remodeling and appetite regulation pathways. Research by Liu and colleagues suggests that dietary BCAA supplementation may exacerbate BCAA accumulation during early IR, while during hyperglycemia and hyperinsulinemia, it does not further elevate BCAA levels but does induce dynamic changes in gut microbiota composition and abundance [126]. Other study has shown that high levels of BCAAs are not the direct cause of obesity and related metabolic disorders; rather, it is the excessive appetite triggered by elevated BCAAs that leads to these conditions [127]. Furthermore, balancing the amino acid profile in the diet by supplementing with tryptophan or threonine can help curb overeating. Therefore, the relationship between dietary BCAAs and circulating BCAAs with FLHS still requires in-depth research.

In summary, bioactive substances derived from gut microbiota metabolism of amino acids (such as indole derivatives, amines, BCAAs) profoundly influence hepatic inflammation, lipid metabolism, and insulin sensitivity. These effects occur through the gut-liver axis, and these substances play a pivotal role in the development and progression of FLHS in laying hens. Notably, the role of BCAAs exhibits complexity and dual effects, involving multifaceted mechanisms such as microbiota remodeling and appetite regulation pathways. Elucidating these microbial metabolites and their mediated signaling pathways will provide a critical theoretical foundation and potential therapeutic targets for understanding FLHS pathology and developing novel intervention strategies (e.g., prebiotics, probiotics, specific amino acid modulation), though their precise mechanisms warrant further investigation.

## Carbohydrate-mediated interactions between the gut microbiota and the host in FLHS

In addition to the mentioned lipids, BAs, and amino acids, carbohydrates also play an important role in the progression of FLHS. Carbohydrates are a pivotal dietary component in the pathogenesis of FLHS in laying hens, influencing both host metabolism and the gut microbiota. Disruptions in carbohydrate metabolism, often exacerbated by high-sugar diets, lead to hepatic lipid accumulation and IR, hallmark features of FLHS [128, 129]. The quantity and type of carbohydrate intake shape the gut microbiota and are profoundly involved in the progression of FLHS. In turn, the gut microbiota ferments carbohydrates to produce metabolites such as SCFAs, which can regulate host energy balance, inflammation, and liver function through the gut-liver axis. This section explores the bidirectional interactions between host carbohydrate metabolism and the gut microbiota in FLHS, focusing on how these interactions contribute to disease progression and potential therapeutic strategies.

### Host carbohydrates regulate gut microbiota involved in FLHS

In FLHS, disruptions in host carbohydrate metabolism, particularly those induced by high-sugar diets, significantly affect the composition and function of the gut microbiota. High-sugar diets increase the concentration of monosaccharides (such as glucose and fructose) in the gut, altering the substrates available to the microbiota and thereby reshaping its ecological niche. These changes not only impact microbial diversity but also regulate host health through pathways such as SCFA production and energy metabolism. Specifically, high-sugar diets can lead to significant changes in the gut microbiota. For example, in goose FLHS, the increase in monosaccharides induced by overfeeding elevates the abundance of Firmicutes and *Lactobacillus* in the jejunum and *Bacteroides* in the cecum [130]. Similarly, in NAFLD patients, high-carbohydrate diets result in marked differences in the abundance of Enterobacteriaceae and Ruminococcaceae compared to healthy individuals [129]. The changes of gut microbiota have important metabolic implications: the increase in Enterobacteriaceae may be associated with excess monosaccharides and intestinal barrier damage, while alterations in Ruminococcaceae affect SCFA production, thereby regulating energy metabolism and fat storage [131]. Additionally, high-sugar diets may cause imbalances in the proportions of Proteobacteria and Bacteroidetes, further amplifying these effects.

Disruptions in host carbohydrate metabolism also affect host metabolism by altering the regulatory functions of the gut microbiota. For instance, high-carbohydrate diets often decrease the abundance of beneficial bacteria like *Lactobacillus* while increasing bacteria associated with IR, such as *Ruminococcus* and *Clostridium* [132]. Conversely, short-term low-carbohydrate interventions rapidly increase the abundance of *Streptococcus* and *Lactococcus*, while reducing the numbers of carbohydrate-fermenting bacteria like *Ruminococcus* and *Bifidobacterium*, leading to decreased SCFA levels [133]. These findings suggest that host carbohydrate metabolism directly shapes the ecological characteristics of the gut microbiota and its regulatory role in host health.

However, current research has limitations. For example, the lack of longitudinal studies tracking microbial changes over time restricts our understanding of the dynamic interactions during FLHS progression. Additionally, there is insufficient analysis of specific metabolic pathways (such as the mechanisms of monosaccharide metabolism) and the unique roles of different carbohydrates (e.g., fructose versus glucose). Future research should prioritize longitudinal studies to capture the dynamic relationship between host carbohydrate metabolism and the gut microbiota. Furthermore, studies focusing on specific dietary carbohydrates and their intervention effects may reveal potential therapeutic strategies to restore microbial balance and alleviate FLHS.

### Regulation of FLHS by microbial carbohydrate metabolites

The gut microbiota metabolizes carbohydrates to produce various metabolites, including SCFAs and succinate, which are then transported to the liver via the gut-liver axis (Fig. 3D). These metabolites regulate lipid metabolism, inflammatory responses, and oxidative stress, thereby influencing the progression of FLHS [134]. The process begins with the breakdown of carbohydrates in the gut by microbial enzymes known as carbohydrate-active enzymes, which include glycoside hydrolases and polysaccharide lyases that decompose complex polysaccharides into oligosaccharides and monosaccharides [135]. This enzymatic activity is exemplified by species like *Bacteroides thetaiotaomicron*, which utilizes its polysaccharide utilization loci to efficiently degrade host mucin glycoproteins and dietary polysaccharides, producing a significant amount of monosaccharides [41]. The resulting oligosaccharides and monosaccharides are further fermented to produce SCFAs, gases such as hydrogen, carbon dioxide, methane, and other metabolites. These fermentation-derived metabolites exert beneficial or harmful effects on FLHS through various pathways. Fermentation primarily involves glycolysis, which converts six-carbon sugars like glucose into pyruvate [136]; the pentose phosphate pathway degrades sugars into five-carbon molecules [137]; and various metabolic routes convert pyruvate into SCFAs.

Different bacteria exhibit varying abilities to metabolize carbohydrates. *Bacteroides*, which are adept at degrading dietary polysaccharides and mucin glycoproteins, primarily produce acetate and propionate [138]. The decrease in *Bacteroides* in the intestines of FLHS laying hens may lead to difficulties in the degradation of polysaccharides and mucin glycoproteins, reducing the production of metabolites such as SCFAs. Firmicutes such as *Roseburia* and *F. prausnitzii* mainly produce butyrate [139], while *A. muciniphila* specializes in degrading mucin glycoproteins, generating acetate and propionate [140].

Moreover, there is a complex network of metabolic cooperation among gut microbes. Some species break down polysaccharides into oligosaccharides or monosaccharides, while others utilize these intermediates for fermentation. For example, the oligosaccharides released after the degradation of xylan by *B. ovatus* can be further utilized by *Bifidobacterium adolescentis*, promoting bacterial growth and the production of lactate and acetate [141]. Similarly, *B. ovatus* produces extracellular inulin-degrading enzymes, facilitating the growth of secondary inulin-degrading bacteria like* B. vulgatus*, which in turn provides a competitive advantage to *B. ovatus* through an unclear mechanism [135]. This intricate cross-feeding balance could affect the availability of metabolites like SCFAs and monosaccharides in FLHS, though such interactions are rarely explored in this context.

Members of the Bacteroidetes phylum, particularly *B. thetaiotaomicron*, build on carbohydrate breakdown by efficiently converting complex polysaccharides into monosaccharides, which play a pivotal role in the initiation and progression of FLHS. The impact of monosaccharides on FLHS varies by type, with fructose exhibiting a higher propensity to trigger FLHS pathogenesis compared to other sugars. Fructose supplementation enhances the villus height to crypt depth ratio in the jejunum, thereby improving intestinal nutrient absorption efficiency. This metabolic shift accelerates hepatic lipid deposition and promotes FLHS development [130]. In geese, excessive intake of monosaccharides (glucose and fructose) induces endoplasmic reticulum stress in hepatocytes, stimulates lipid synthesis, suppresses fatty acid β-oxidation, and impairs lipid transport, collectively driving FLHS onset [142]. Geng et al. demonstrated that combined treatment with high glucose concentrations (25 mmol/L and 50 mmol/L) and palmitate synergistically induce endoplasmic reticulum stress in primary goose hepatocytes, exacerbating intracellular lipid accumulation [143]. Prolonged high-glucose supplementation in geese similarly activates hepatic endoplasmic reticulum stress, promotes IR, and ultimately leads to FLHS manifestation.

In contrast to the potentially harmful effects of monosaccharides, SCFAs, the main fermentation products of dietary carbohydrates, play a protective role in FLHS [144]. For example,* B. uniformis* possesses numerous CAZy genes related to carbohydrate metabolism and can convert complex polysaccharides and cellulose into acetate, which exerts anti-inflammatory effects, prevents diet-induced inflammation, and improves liver inflammation through IL-22 signaling [145, 146]. However, not all metabolites are uniformly beneficial. Succinate is another important product of carbohydrate metabolism by the gut microbiota. It serves both as a precursor to SCFAs and as an independent signaling molecule with bidirectional effects on liver metabolism. Bacteria such as *Bacteroides*, *Prevotella*, *Succinivibrio*, *Ruminococcus*, and *Veillonella* ferment carbohydrates to produce succinate or its salts [147]. Research has shown that *Prevotella copri* produces succinate, which modulates hepatic lipid metabolism and antioxidant activity, thereby lowering blood lipid levels and reducing liver fat content in FLHS laying hens [147]. Similarly, *Parabacteroides distasonis* generates succinate and, by interacting with fructose-1,6-bisphosphatase, activate intestinal gluconeogenesis to lower hyperglycemia while improving lipid metabolism [148].

However, some studies have also documented negative effects of succinate. Serum concentration of succinate is significantly higher in obese individuals and may promote the development of fatty liver by activating Kupffer cells and hepatic stellate cells [149]. In NAFLD models, succinate promotes hepatic TG deposition by inhibiting fatty acid oxidation. Further studies indicate that this effect is linked to succinate’s suppression of FGF21, and its inhibitory impact on FGF21 expression is dependent on the AMPK/PPARα axis [150]. Mills et al*.* further elucidated this mechanism that succinate drives inflammation through succinate receptor 1, which is expressed on hepatic stellate cells and resident macrophages, thereby exacerbating fatty liver disease [151].This duality suggests that the specific impact of succinate depends on factors such as dosage, individual metabolic state, and the composition of the gut microbiota. Beyond these metabolites, other microbial products also contribute to improving fatty liver. For example, *P. distasonis* can utilize inulin to produce odd-chain fatty acids like pentadecanoic acid, which help restore the intestinal barrier and lower LPS levels, thereby alleviating hepatic inflammation and steatosis [152].

In summary, microbial carbohydrate metabolites significantly influence FLHS progression by modulating host energy balance, BA metabolism, and inflammatory status. SCFAs like acetate, propionate, and butyrate enhance insulin sensitivity, reduce inflammation, and improve lipid metabolism, potentially alleviating liver damage in FLHS. Conversely, monosaccharides like fructose and succinate can exacerbate FLHS under certain conditions, highlighting their dual roles. Despite these insights, current knowledge remains incomplete due to limited mechanistic studies on metabolite-host interactions and unidentified beneficial or detrimental compounds, underscoring the need for further research.

## Conclusion and future perspectives

The pathogenesis of FLHS involves complex interactions between the gut microbiota and host metabolites. This review has systematically discussed how lipids, BAs, amino acids, and carbohydrates mediate the interactions between the host and gut microbiota, regulate hepatic inflammation, oxidative stress, and lipid accumulation, and contribute to the progression of FLHS. Microorganisms such as *Bacteroides*, *Lactobacillus*, and *A. muciniphila* may be key targets for regulating FLHS, primarily by modulating the production of their metabolites. Key findings highlight the protective role of SCFAs in promoting fatty acid oxidation and reducing inflammation, and butyrate can serve as a key metabolite for regulating FLHS. Meanwhile, BAs exhibit dual functions by modulating microbial ecology and maintaining host metabolic homeostasis. On the other hand, amino acid metabolic disorders worsen disease progression through immune dysregulation and oxidative stress, while disrupted carbohydrate metabolism exacerbates hepatic glycolipid metabolic disorders and accelerates the occurrence of IR. These metabolites form a bidirectional regulatory network through the gut-liver axis, underscoring the pivotal role of the gut microbiota in FLHS.

Despite recent advances in our understanding of FLHS pathogenesis, many questions remain. The full spectrum of microbiota and its metabolic products in FLHS have yet to be completely elucidated, particularly regarding how specific microbial species regulate host lipid homeostasis. Moreover, the causal relationship between FLHS and gut microbiota imbalance is still unclear. It remains critically unclear whether the observed microbial imbalance is a primary driver that initiates hepatic lipid accumulation and inflammation, or merely a secondary consequence of the host's established metabolic dysfunction (e.g., altered bile acid secretion or inflammation) shaping the gut environment. In addition, intervention strategies based on the microbiota and its metabolites remain in the exploratory stage. The effectiveness of approaches such as probiotics, synthetic metabolites, and targeted microbiota modulation has yet to be validated in large-scale clinical trials, and there is still a long way to go before they can be developed into drugs. And their underlying mechanisms require further in-depth investigation. Future studies should focus on: (1) employing combined approaches such as metabolomics, microbiomics, and transcriptomics to dynamically track changes in the gut microbiota and its metabolites during FLHS progression and construct a comprehensive metabolic regulation map; (2) utilizing germ-free animals or fecal microbiota transplantation, researchers can directly assess whether gut microbiota is sufficient to induce or reverse the FLHS phenotype, thereby providing compelling evidence for a causal relationship involving the microbiota; (3) investigating the molecular interactions between specific microbial taxa (such as *Bacteroides* and *Akkermansia*) and host receptors like FXR and TGR5 to elucidate key signaling pathways; and (4) developing targeted interventions based on probiotics, prebiotics, or metabolite supplementation (including SCFAs and BA derivatives) to evaluate their effectiveness in preventing and treating FLHS and optimize delivery methods for clinical application. Given the similarities in pathological mechanisms between FLHS in laying hens and NAFLD in humans, future research may also benefit from advances in NAFLD studies to develop more precise strategies for FLHS prevention and treatment. However, extrapolations must be approached with caution due to inherent differences. NAFLD is more prevalent in human males, whereas FLHS occurs primarily in female poultry under estrogen-driven metabolic demands [153, 154]. These disparities suggest divergent etiologies, including species-specific hormonal influences. To bridge this gap, comparative studies integrating sex-specific multi-omics data (e.g., estrogen-modulated gut microbiota profiles) are essential. Such prudence will ensure that NAFLD-derived interventions, like BA modulators, are rigorously tested in avian models before application to FLHS.

## Data Availability

Not applicable.

## Abbreviations

AMPK: Adenosine 5'-monophosphate-activated protein kinase
BAs: Bile acids
BCAAs: Branched-chain amino acids
BSH: Bile salt hydrolase
CLA: Conjugated linoleic acid
DCA: Deoxycholic acid
FGF: Fibroblast growth factor
FLHS: Fatty liver hemorrhagic syndrome
FXR: Farnesoid X receptor
IR: Insulin resistance
LCA: Lithocholic acid
NAFLD: Non-alcoholic fatty liver disease
NF-κB: Nuclear factor kappa-B
PC: Phosphatidylcholine
PPARs: Peroxisome proliferator-activated receptors
SCFAs: Short-chain fatty acids
SREBP-1c: Sterol regulatory element-binding protein-1c
TG: Triglyceride
TGR5: G protein-coupled bile acid receptor 1
